# Can deep learning beat numerical weather prediction?

**DOI:** 10.1098/rsta.2020.0097

**Published:** 2021-02-15

**Authors:** M. G. Schultz, C. Betancourt, B. Gong, F. Kleinert, M. Langguth, L. H. Leufen, A. Mozaffari, S. Stadtler

**Affiliations:** Jülich Supercomputing Centre, Forschungszentrum Jülich, Germany

**Keywords:** numerical weather prediction, machine learning, deep learning, weather AI, spatiotemporal pattern recognition

## Abstract

The recent hype about artificial intelligence has sparked renewed interest in applying the successful deep learning (DL) methods for image recognition, speech recognition, robotics, strategic games and other application areas to the field of meteorology. There is some evidence that better weather forecasts can be produced by introducing big data mining and neural networks into the weather prediction workflow. Here, we discuss the question of whether it is possible to completely replace the current numerical weather models and data assimilation systems with DL approaches. This discussion entails a review of state-of-the-art machine learning concepts and their applicability to weather data with its pertinent statistical properties. We think that it is not inconceivable that numerical weather models may one day become obsolete, but a number of fundamental breakthroughs are needed before this goal comes into reach.

This article is part of the theme issue ‘Machine learning for weather and climate modelling’.

## 1. Introduction

The history of numerical weather prediction (NWP) and that of machine learning (ML) or artificial intelligence (for the purposes of this paper, the two terms can be used interchangeably) differ substantially. First, manual NWP was attempted by Lewis Fry Richardson in Britain in 1922, and early computer-aided weather forecasts were produced in 1950 [1]. These were soon followed by operational weather forecasts in Sweden, the USA and Japan. While there has been steady progress in the development of NWP (cf. [2]), the history of ML (cf. [3]) has been more disruptive: the first neural network (NN) was proposed in 1943 by McCulloch & Pitts [4]. The field expanded until the early 1960s, when the existing algorithms proved inefficient and unstable. The invention of backpropagation in 1970 [5,6] led to a second wave of ML applications as it became possible to build more extensive NNs and train them to recognize nonlinear relationships in data (e.g. [7]). Even though the development of ML algorithms continued, the enthusiasm about them soon dwindled again, because they rarely showed significant performance gain, and computing resources were not sufficient to solve larger problems. Furthermore, big amounts of labelled data which are mandatory for most data-driven ML approaches were hard to come by (note that this was before the advent of the world wide web). Three significant developments around 2010 started the third wave of artificial intelligence, which continues to the present: computing capabilities were vastly expanded due to massive parallel processing in graphical processing units (GPUs), convolutional neural networks (CNN) allowed much more efficient analysis of massive (image) datasets, and large benchmark datasets were made available on the internet. Highly complex NNs with greater than 10^6^ parameters enabled a breakthrough in image recognition [8], soon followed by remarkable success stories in speech recognition [9], gaming [10,11], and video analysis and prediction [12,13]. Today’s NNs are often deep networks with greater than 10 layers, and the research field which develops such NNs and the associated methods for training and validation is called deep learning (DL).

**Figure 1.**
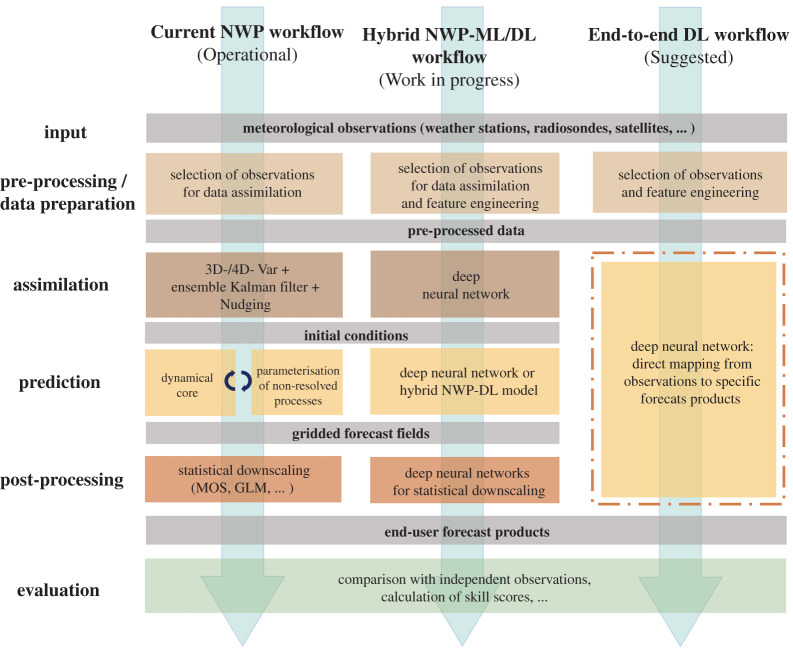
Idealized workflows of current numerical weather prediction (left), next-generation weather prediction with individual components substituted or augmented by ML and DL techniques (centre), and a purely data-driven DL weather forecasting system (right). (Online version in colour.)

The weather and climate research community is increasingly aware of modern DL technologies and tries to adopt them to solve specific data analysis, numerical modelling and post-processing problems in the context of NWP. Nevertheless, as the workshop on ‘Machine learning in weather and climate’ (Oxford, September 2019) has also shown, there are still reservations about DL in this community. Two core arguments in this regard are the lack of explainability of deep NNs and the lack of physical constraints. Furthermore, some scepticism prevails due to the fact that researchers have experimented with rather simple NNs which were clearly unsuited to capture the complexity of meteorological data and feedback processes, but then extrapolate these results to discredit any NN application including the much more powerful DL systems. In their review of ‘Deep learning and process understanding for data-driven Earth system science’, Reichstein *et al.* [14] argue that traditional ML approaches might not be optimally suited to address the specific data challenges posed by Earth system data. They suggest that ‘deep learning methods are needed to cope with complex statistics, multiple outputs, different noise sources and high-dimensional spaces [of Earth system data]. New network topologies that not only exploit local neighbourhood (even at different scales) but also long-range relationships (for example, for teleconnections) are urgently needed, but the exact cause-and-effect relations between variables are not clear in advance and need to be discovered.’ As modern DL methods begin to deliver such concepts, we take this opportunity to expand on the analysis of [14] and explore the applicability of such methods to the NWP workflow (figure 1, left column).

While considerable work is undertaken to substitute specific parts of the NWP workflow with DL approaches (figure 1, central column), in this paper, we take a bold step forward and address the question of whether it is possible to replace all core parts of the NWP workflow with one deep NN, which would take observations as input and generate end-user forecast products directly from the data (figure 1, right column). Such approaches have been investigated for the specific application of wind speed and power predictions (cf. [15]), and there is at least one study, which successfully developed an end-to-end workflow to forecast multiple weather variables from the data of the US weather balloon network [16]. However, most of these studies were restricted to short-term forecasts and a few individual target sites, and none has yet attempted to explore the wealth of combined meteorological observations from the plethora of instruments and sensors, which is routinely used in operational weather forecasts.

The goal of this paper is therefore to review the NWP workflow from a DL perspective and analyse the specific requirements and conditions of weather forecasting in light of current DL theory and practices. It is structured as follows: §2 gives a brief overview about the major developments and state of the art of NWP including aspects of data assimilation (DA) and model output processing. It is followed by §3 which surveys the literature on fundamental ML and DL developments and their application to weather and climate research. Section 4 discusses several fundamental aspects of meteorological data and other requirements of weather forecasts and points to corresponding solutions in DL research where these exist. Section 5 reflects on two aspects which are relevant for both weather forecasting and DL, but where we find different best practices in both domains. These aspects are data preparation and model evaluation. In §6, we reflect on the issues of physical constraints and system-wide forecast consistency in a DL framework. Section 7 discusses the state of the art with respect to estimating forecast uncertainties. Finally, §8 presents conclusions. We hope that this article will lead to a better understanding between ‘machine learners’ and ‘weather researchers’ and thus contribute to a more effective development of DL solutions in the field of weather and climate.

## 2. State-of-the-art numerical weather prediction

Modern weather prediction relies extensively on massive numerical simulation systems which are routinely operated by national weather agencies all over the world. The associated process chain to generate these numerical weather forecasts can be divided into several steps which interact closely with each other (figure 1, left column).

In order to retrieve the initial state of the Earth system (atmosphere, soil and ocean), a great variety of meteorological observations are collected all over the world. In addition to classical weather and radiosondes stations, aircraft measurements and remote sensing products (such as radar and satellite observations) have become an integral part of the global observation network (e.g. [17]). Although millions of different direct and indirect measurements are obtained every day, these observations are still not sufficient to describe the complete state of the atmosphere and other Earth system components with which the atmosphere exchanges energy or mass.

At this point, DA comes into play. The central task of DA is to fill the gap between the incomplete, heterogeneous, and scattered observations and the initial value fields which are required by the NWP models. To achieve this, the observation data must be projected onto the discrete model grid, interpolated in time and adjusted to be consistent in terms of state variables (e.g. temperature, pressure, wind etc.). DA also has to take into account measurement errors such as biases between different space instruments or malfunctions of individual ground-based sensors. The obtained initial state of the Earth system after the DA step therefore constitutes an optimized estimate of the real conditions (e.g. [18]).

Given the initial conditions, the NWP model can perform a simulation of atmospheric processes. By solving numerically the coupled partial differential equation system describing the atmosphere in terms of momentum, mass and enthalphy (the Navier–Stokes equations), the future atmospheric state is obtained in each model grid cell. Processes occurring at scales smaller than the model grid size are captured by empirical parameterizations. The direct model output constitutes the first forecast product of the NWP workflow. In contemporary global NWP models, the grid boxes cover an area of several square kilometres.

In order to arrive at finer scale end-user forecast products, a post-processing step is added to the NWP workflow. The outcome of such post-processing can cover a variety of forecast products starting with the conversion of the vertical axis from sigma-coordinates to pressure levels or geometric height (above mean sea level) or bias corrections. Statistical methods are applied to remove systematic biases of the NWP output and to incorporate local scale adjustments (statistical downscaling). Furthermore, limited-area models which allow for finer grid spacings (Δx∼O(1–5 km) compared to Δx O∼(10 km) in global models) provide added value on forecasting meteorological features on finer scales. The output of ensemble simulations can be used to estimate forecast uncertainties which are of major interest especially for high impact weather situations, or for the renewable energy sector [19,20] (see also §7).

Over the past decades, the ability of NWP models to predict the future atmospheric state has continuously improved. Contemporary global NWP models are not only able to predict the synoptic-scale weather pattern for several days, but they have also reached remarkable accuracy in forecasting end-user relevant meteorological quantities such as the 2 m temperature and regional-scale precipitation events. For instance, the deterministic forecasts of the Integrated Forecast System provided by the European Centre for Medium-Range Weather Forecasts maintains an anomaly correlation coefficient of the 500 hPa geopotential height of 80% for about 7 days, while the root-mean square error for 2 m temperature predictions of 72 h forecasts is close to 2 K [21]. Larger scale high-impact events such as hurricane tracks can be predicted with an accuracy of 150 km up to 4 days in advance [22].

The increasing success of operational NWP models is due to improvements of all the steps involved in the NWP workflow and new capabilities of the global Earth observation system. In the following, we briefly highlight a couple of important developments which led to significant enhancements of forecast quality. A detailed review of recent advances in the NWP process chain is beyond the scope of this article.

While *in situ* observations (weather stations and radiosondes) have a long history in observing the Earth’s atmosphere, fundamental improvements in the spatiotemporal coverage of observations has been achieved with the help of satellite data over recent decades (e.g [23]).

Nowadays, several geostationary and polar-orbiting satellites deliver a great variety of data products (such as temperature and humidity profiles, soil moisture and atmospheric motion vectors). Satellite data are particularly valuable as they provide information on the atmosphere above the ocean and uninhabitated areas where conventional measurements are hard to come by. However, measurements from commercial aircraft (e.g. [24]) and radar observations (e.g. [25,26]) have also contributed to better constraining the initial state of NWP models.

The ability of DA systems to make use of the manifold, diverse observations has seen continuous improvement due to algorithmic developments. Current DA systems are primarily based on three- or four-dimensional variational approaches (3D-Var and 4D-Var, respectively) and on ensemble methods (commonly Kalman filter). In the 3D-Var approach, a single deterministic state is estimated by minimizing a cost function which generally consists of the three terms background, observation, and model error. 4D-Var additionally captures observation changes in time (see [18] for more details).

In order to obtain a loss function which can be optimized with reasonable efficiency, the model and observation operators have to be linearized. This can lead to forecast errors, in particular if the NWP model contains discontinuous parameterizations [27]. Another simplification of the variational DA approach is the *a priori* definition of the uncertainties of the state vector **X** which leads to a static background error covariance matrix. By contrast, an ensemble approach allows for dynamic estimation of the probability density function of **X**. The Ensemble Kalman Filter approach makes use of such an estimation which then results in a non-static, i.e. flow-evolving background error covariance matrix. A disadvantage of classical ensemble methods is that they are only conditioned on past measurements [28]. Therefore, leading meteorological centres have started to establish combinations of the variational approach with ensemble approaches such as the 4D-EnvVar DA method. The quality of ensemble DA depends on the number of ensemble members which is typically restricted to a relatively small number due to computational reasons. Therefore, so-called hybrid DA systems have been developed which include climatological error information in order to lessen the sensitivity to undersampling [29].

NWP model improvements can in part be related to resolution enhancements. The continuous refinement of the grid spacing has also required re-formulating the dynamical cores of NWP models where the discretized Navier–Stokes equations are solved. Simulating the atmosphere on kilometre-scale comes along with the demand of highly parallelizable algorithms of the dynamical core [30]. Since (classical) global spectral transform models are less suited for such a requirement, finite-difference or finite-volume discretizations on platonic solids projected on the sphere (e.g. icosahedral [31] or cubed-sphere grids [32]) have been developed over the previous decade. Simultaneously, remarkable progress has been achieved in designing discretization approaches which enable the grid-scale dynamics to follow the conservation laws of energy, enthrophy and mass [33,34] while also minimizing the need for numerical filters to suppress artificial numerical modes [35]. An extensive overview of contemporary dynamical core architectures can be found in [32].

In addition to the improvement of dynamical cores, further gains in accuracy have been achieved by fine-tuning physical parameterizations which are mandatory to represent atmospheric processes that cannot be captured by the grid-scale thermodynamics. Among others, these parameterizations encompass the representation of (deep) convection, turbulent mixing, smaller-scale atmosphere-land/ocean coupling, the representation of cloud microphysics and radiative transfer. Advances in capturing the diurnal cycle of convection (e.g. [36,37]), the turbulent transports in the planetary boundary layer (e.g. [38]) and in simulating the bulk properties of hydrometeors (e.g. [39]), i.e. clouds and precipitation, are only a small sample of recent progress in tuning physical parameterization schemes.

## 3. Deep learning in weather research

The increased computational power, the availability of large datasets, and the rapid development of new NN architectures all contribute to the ongoing success of DL. Some of these new NN can solve certain ML tasks much more efficiently than the classical fully connected, feed-forward networks. One especially successful concept, which has been widely applied, is convolutional neural networks [40] (CNN), where a stack of small-sized filters with few trainable parameters is applied to images or other gridded data to extract coarser scale features. CNNs have been used in weather and climate applications, where the NN was trained to recognize spatial features, for example in the analysis of satellite imagery [41] or weather model output [42].

The family of recurrent neural networks (RNN) was designed specifically for the learning of time-dependent features (i.e. text and speech recognition). More advanced RNN architectures are long short-term memory (LSTM) nodes [43,44] and gated recurrent units (GRU, [45]). LSTM and GRU cells can be embedded in more complex neural network architectures. For example, the combination of a normal CNN with LSTM yields the so-called ConvLSTM network [46].

Two more recent DL concepts are variational auto-encoders (VAE) [47] and generative adversarial networks (GAN) [48]. Both of these are so-called generative models, i.e. they learn the data distributions from training samples and use generators to produce novel samples which match the characteristics of the training data. They are widely used in different applications such as image-to-image translation [49], super-resolution image generation [50], in-painting [51], image enhancement [52], image synthesis [53], style transfer and texture synthesis [54] and video generation and prediction [12,13]. VAEs use an encoder to project the high-dimensional data with posterior distribution into a latent space with lower dimensionality. This latent space is then sampled by a decoder to reconstruct the original feature space in all dimensions. For further information on VAE, we refer to [47,55]. In GANs, the competition of two NNs is used to improve on image generation during training. One network is optimized to generate realistic images, while a second one is trained concurrently to discriminate between generated and real images. Typically, both VAE and GAN-based architectures are coupled to multiple convolutional layers for capturing the semantic features of the input data and represent them with fewer dimensions. Examples are PixelVAE [56], DCGAN [57], sinGAN [58] and SAVP [13]. It is a general tendency in DL research that new NN architectures are composed of many building blocks which are themselves substantially large DL networks. The problem-complexity which can be addressed with modern DL networks is already quite substantial. The largest NNs have several million degrees of freedom which is comparable to operational NWP models.

ML as ‘an approach to data analysis that involves building and adapting models, which allow programs to learn through experience’ has been employed by meteorologists for a long time, for example in curve fitting, linear regression, or DA (see §2). However, in this article, we focus on ML in a narrower sense, i.e. involving NNs and in particular modern DL.

First studies employing NN for meteorological and air quality applications appeared during the 1990s [59–61]. These studies used multi-layer-perceptron architectures with typically three layers to analyse and forecast time series at individual station locations. Later, other simple semantic network techniques were used for post-processing and prediction optimization of NWP output [62,63], and as surrogate models for different parameterization schemes in climate models [64,65].

It took a few years before the weather and climate research community started to pick up modern DL concepts and began to explore their use in NWP and other environmental applications. Table 1 lists various state-of-the-art DL architectures and their first applications in weather and climate research. A couple of examples are briefly described below. A review of ML in remote sensing can be found in [41]. Zhou *et al.* [83] and Denby *et al.* [84] used a CNN for classification of weather satellite images, while Xu *et al.* [85] used a combination of GAN and LSTM for prediction of cloud images. Based on the concept of video prediction, various types of networks were used for short-term prediction of sky images and radar images [46,79,81]. There have also been some attempts to produce data-driven weather forecasts, for example by Dueben & Bauer [86], who used a multi-layer perceptron, or Grover *et al.* [16], who constructed a three-stage model consisting of boosted decision trees, a dynamic Gaussian Process model, and a deep belief network consisting of restricted Boltzman machines [77,78]. The study of Wandel *et al.* [87] could be regarded as a first step towards replacing the dynamical core of a numerical weather model as they demonstrate unsupervised learning of the full incompressible Navier–Stokes equations on a Eulerian, grid-based representation. Gong *et al.* (A. B. Gong, unpublished manuscript, 2020), experimented with a state-of-the-art video prediction model and tried to use it for 2 m temperature predictions over 10 h.

**Table 1.** State-of-the-art neural network architectures and their application in weather and climate research. Entries marked with * denote hybrid NN architectures.

architecture	introduced for the first time (original references)	early weather and climate applications (current state in July 2020)
convolutional neural network (CNN)	AlexNet (Alex *et al.* 2012 [8])	VGG (Shi *et al.* 2018 [66])
	VGG (Simonyan & Zisserman, 2013 [67])	ResNet (Pothineni *et al.* 2019 [68])
	ResNet (He *et al.* 2015 [69])	Vgg, ResNet (Wen *et al.* 2020 [70])
	GoogleLeNet (Szegedy *et al.* 2015 [71])	Inception blocks (Kleinert *et al.* 2021 [72])
long short-term memory network (LSTM)	RNN (Bengio *et al.* 1994 [73])	LSTM (Gómez *et al.* 2003 [74])
	LSTM (Hochreiter and Schmidhuber, 1997 [43])	LSTM (Qing & Niu, 2018 [75])
		PhyDNet (Le Guen, 2020 [76])
deep belief network (DBN)	Smolensky, 1986 [77], Hinton & Salakhutdinov [78]	Grover *et al.* 2015 [16]
variational autoencoder (VAE)	Vanilla VAE, 2013 [47]	None
generative adversarial neural network (GAN)	Vanilla GAN (Goodfellow *et al.* 2014 [48])	MD-GAN (Xiong, 2018 [79])
		conditional GAN (Schmidt *et al.* 2020 [80])
convolutional long short-term memory network (ConvLSTM)	convLSTM (Shi *et al.* 2015 [46])	ConvLSTM* (Shi *et al.* 2015 [46]),
		PredRNN (Wang *et al.* 2017 [81]); MIM (Wang *et al.* 2019 [82])
stochastic adversarial video prediction (SAVP*)	SAVP (Lee *et al.* 2018 [13])	Gong *et al.* (A. B. Gong, unpublished manuscript, 2020)

## 4. Challenges of end-to-end deep learning weather prediction

The studies that were cited in the previous section already demonstrate that DL concepts can be successfully applied to problems related to weather forecasting. However, the few existing attempts to replace the entire NWP workflow with a DL system have been limited to short-term forecasting (up to 24 h or less) or used a rather limited subset of the available meteorological data. In this section, we discuss a number of challenges which need to be overcome before a complete end-to-end DL weather forecasting system can deliver results of comparable quality as current NWP.

**Figure 2.**
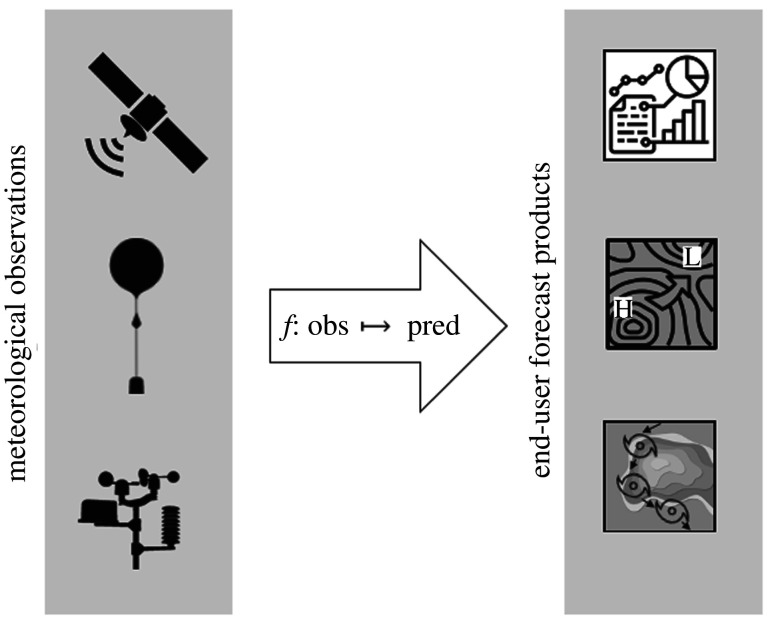
Schematic view of the principal task of weather prediction, i.e. a mapping from diverse observation data to specific forecast products.

Weather forecasting is essentially a prediction of spatiotemporal features based on a diverse array of observations from ground-based, airborne and satellite platforms. If we treat the core part of the NWP workflow (figure 1), i.e. DA, model forecast and output post processing, as one entity, then a weather forecast can be described as a function, which maps observation data to a final forecast product (figure 2). The forecast product can be a map of a specific weather variable (e.g. temperature), a time series of one or more variables at a specific location or aggregated over a region, some aggregate statistics of a specific variable over a given time range, a (categorical) warning index, etc. With current NWP, we are used to employing one forecasting system (which may well have several components) to derive the whole set of forecast products which are requested by end users, or needed for the system evaluation and further improvement. By contrast, DL applications excel if they can focus on a specific task, i.e. a reasonably small set of target variables. Therefore, an end-to-end DL weather forecast as depicted in the right column of figure 1 would likely consist of several deep NNs which would be trained individually on specific subsets of forecast products.

Advantages of such a DL weather forecasting system could be the intrinsic absence of model bias (because the system would be trained to reproduce the target values) and the possible savings of computational resources. Once NNs are trained, they can very efficiently calculate forecasts with new data. Forward propagation in NNs consists only of fast add and multiply operations. Therefore, even NNs with O(10^8^) parameters (i.e. similar complexity as contemporary NWP models) can be expected to use far less computing resources than current numerical models. The determining factors of the required computational resources in an end-to-end DL weather forecasting system are the necessary training cycles and the data processing. The former will depend on the learning approach (e.g. lifelong learning [88] requires regular re-training of some NN components) and on the success of transfer learning [89] concepts (i.e. whether it is possible to re-use NNs trained in one region of the globe for weather forecasts in another region). Data processing is a challenge which also limits further scaling of NWP models and other applications on current and next generation supercomputing systems (see [90]). At present, it is impossible to predict how much computing time, if any, could be saved if all weather forecasting would be based on DL. A fair comparison should always consider the entire weather prediction workflow and the whole range of forecast products that shall be generated.

**Figure 3.**
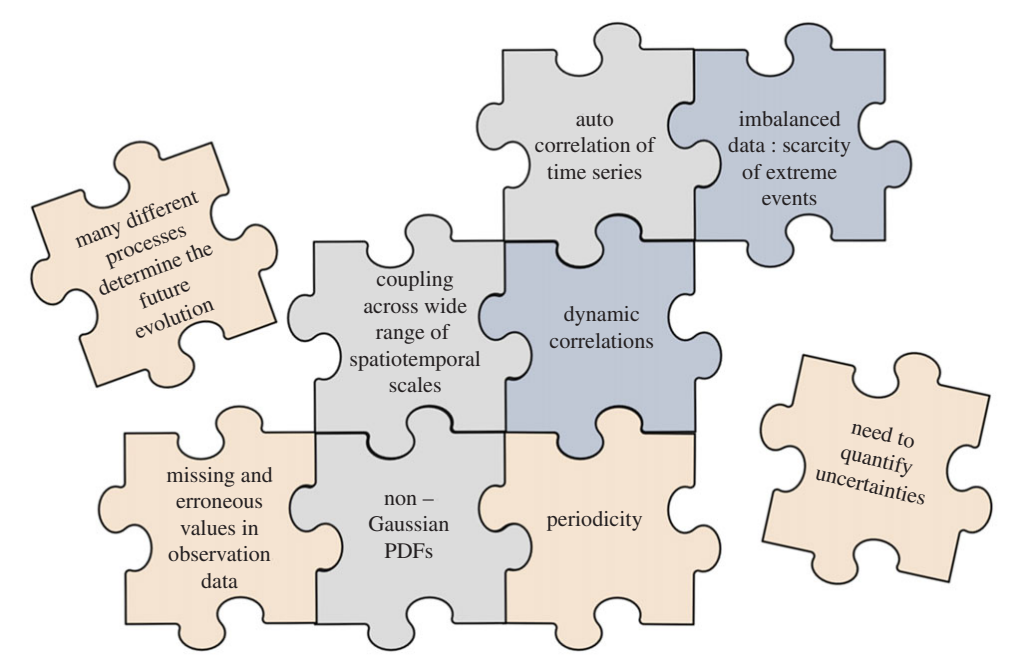
Graphical summary of the inter-connected DL challenges imposed by properties of meteorological data and the complexity of the weather forecasting problem. The colours of the jigsaw pieces are arbitrary. (Online version in colour.)

In the following, we discuss a number of challenges for end-to-end DL weather predictions, which are mostly a consequence of the specific properties of meteorological data and the complexity of the atmosphere and its interactions with other Earth system compartments. These challenges are graphically summarized in figure 3. As will be seen, many of these challenges also appear in other DL contexts, and the DL community has begun to develop solutions for these problems. Nevertheless, there are no systems in place which can cope with all of these challenges together.

The success of DL methods hinges on a good understanding of relevant data properties. Meteorological variables can be described with different cumulative distribution functions or corresponding probability density functions. Some variables (e.g. temperature) are nearly normally distributed, while others (e.g. precipitation and cloud droplet size) might be better approximated by gamma or beta distributions [91]. The fraction of cloud cover is often reported in eighths and therefore needs to be treated as a discrete variable. Ignoring the different properties of meteorological data can cause erroneous results when they are not accounted for in a statistical analysis or forecasting procedure. This is particularly relevant as some DL methods (e.g. the Bayesian approach by [92]) make implicit assumptions about the frequency distribution of variables.

Meteorological features can show dynamic behaviour on a wide range of spatiotemporal scales, and the quality of the weather forecast is influenced by phenomena on many different scales [93]. Consider sea ice as an example, which may change little during the time of a typical forecast lead time, but whose mid- to longer-term variations can have a profound influence on the local and non-local atmospheric state [94]. Multiple spatial scales have been investigated in the context of video prediction (e.g. [12,58]).

Related to the interaction of scales, the spectra of energy and momentum are important aspects in meteorology, but such spectra do not play a similar role in most mainstream DL applications. However, spectral transformations have already been used in DL applications (e.g. [9] in the context of speech recognition). First attempts at using such concepts in ML weather applications [95–98] were limited to simple NNs and limited complexity datasets.

Many meteorological features vary periodically, although there can be large variability between cycles. This periodicity is induced by orbital parameters and the Earth rotation together with various solar cycles. As shown in Ziyin *et al.* [99], NNs generally have difficulties extrapolating periodic features correctly. However, as the authors show, replacing common monotonic activation functions with functions which include a periodic term can solve such problems and produce, for example, better temperature forecasts.

Meteorological variables are correlated in space and time, and these correlations change with time [16]. For example, temperatures at different altitudes may exhibit very similar patterns (possibly with a time lag) in a well-mixed boundary layer (i.e. summer, daytime), while different vertical levels can be almost completely decoupled during an inversion (often during winter or at night). This can also be seen in larger scale features such as tropical cyclones, Rossby waves, fronts and (organized) convection. While we are not aware of a publication which addresses this issue in the context of DL with weather data, there are other DL studies which demonstrate that it is possible to cope with such correlations (e.g. [100]).

A related property of meteorological features is auto-correlation. While auto-correlation in a way simplifies the forecasting task (at least on short time scales), it imposes the risk of over-estimating the forecasting capabilities of a DL model, in particular if this is not factored into the data preparation and model evaluation (see §5).

Meteorological features may appear and vanish on time scales much shorter than the forecast range. Prominent examples are the triggering of convective cells or the transition between convective and relief precipitation in the presence of orography. An NWP model has some skill in predicting such features, because it can diagnose their potential occurrence from relations between other variables. In principle, such complex relations may be decipherable by NNs as well. However, we believe that this will require additional measures to make the NN aware of such relations. Such measures could be feature engineering (i.e. the calculation of derived properties from combinations of input variables) or the implementation of physical constraints (see §6).

Another challenge for a DL weather forecast application is the scarcity of extreme events, which are, however, very important to get right as extreme weather phenomena have the largest impact on civil safety and economy. For example, to accurately predict heavy precipitation events (>25 mm h^−1^) over Germany, the DL model must be trained with less than 10 episodes at any given location during a full decade [101]. A few studies have touched upon the subject of ML and extreme climate events: Vandal *et al.* [102] find that complex DL models have more problems in capturing extremes than classical statistical downscaling models, whereas O’Gorman *et al.* [103] state that their model captures extremes quite well without the need for special training on these cases. While the subject of classifying imbalanced data has received considerable attention (cf. [104]), there appears to be little research on dealing with imbalanced sample sizes in regression problems [105]. In contrast to standard DL algorithms, humans have acquired the ability to learn from isolated extreme events because they pay special attention to extraordinary occurrences in their environment and quickly generalize to other situations [106]. Some studies have explored the possibility to have deep neural networks learn extreme events in a similar way. For example, Li *et al.* [107] implemented one-shot learning of object categories by using prior knowledge learned from other training samples. Lake *et al.* [108] developed ML methods within Bayesian program learning (BPL) to mimic human capabilities of learning visual recognition from a few samples.

Finally, other critical aspects related to meteorological observation data are the frequent appearance of missing values and the possibility of input data errors and biases. Current DA procedures often include a substantial code base for filtering or interpolating missing values, blacklisting observations, and monitoring of biases and their evolution over time. Similar issues occur in various application areas of DL. For example, Smieja *et al.* [109] have dealt with the issue of missing data values, and Žliobaité [110] and Lu *et al.* [111] investigated the problem of drifting biases (known as concept drift in the ML community). A recent example of a DL application working on meteorological satellite data with missing values is Barth *et al.* [112].

## 5. Data preparation and model evaluation

In this section, we discuss two aspects of ML in weather and climate, which we have found to be important in our practical experience and where best practices differ between the meteorological and ML communities. These are data preparation and model evaluation. This discussion may shed some light upon the reasons why it has been difficult for the DL community to tackle weather data problems and why, conversely, the meteorological community has been cautious to adopt DL in their research and for routine weather analyses and forecasts.

All ML techniques are data-driven. Therefore, proper selection and preparation of data are essential to gain good and generalizable results. Data selection should aim to capture the full variability of the predictor variables, avoid too much redundant information, and allow the network to capture relations among variables, from which a prediction can be made. For the sake of brevity, we will not discuss data selection in more detail, but instead focus on data preparation aspects in the following.

Modern supervised ML studies generally divide the available data into three different datasets to train, develop and evaluate an ML model [113]. The training set is the largest and is used to update the model parameters by back propagation or other learning algorithms. The second set, which is often referred to as the validation or development set, is used exclusively for hyper-parameter tuning. The hyper-parameters, i.e. number of layers, type of layer, activation function, loss function, learning rate etc. are set manually by the model developer. A key target of this hyper-parameter tuning is the optimization of the network’s generalization capabilities to ensure that the network will function well on previously unseen data. Both parameters and hyper-parameters are essential for building a suitable DL model. The third dataset is the test set, a collection of previously unseen data which is used to evaluate the network after the tuning to assess the true generalization capability of the network. The three datasets should be independent of each other, but at the same time they should reflect the same statistical distribution. Therefore, one has to be careful how to split the data before starting to train a new network, especially if, as in meteorological time series, the data are auto-correlated.

**Figure 4.**
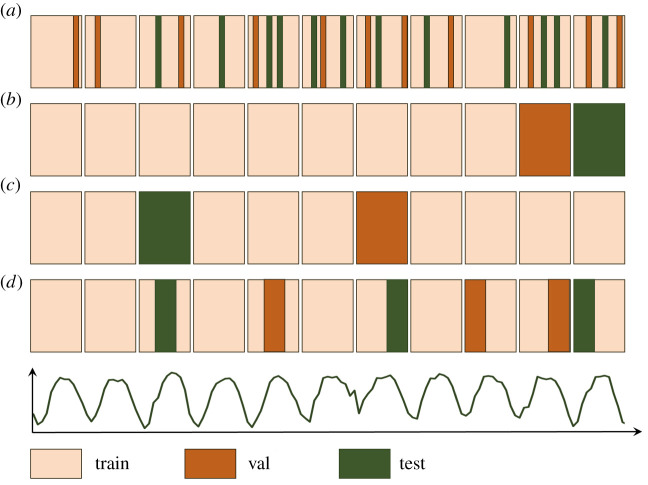
Different train-dev-test splitting strategies for meteorological data with periodic features as indicated in the conceptual time series at the bottom of the figure. Every sand coloured block stands for 1 year of data. Case (*a*) depicts random sampling as is commonly applied in many DL applications. Cases (*b*–*d*) show different variants of random block sampling, which avoid spurious correlations between the train, val and test sets, if the block length chosen is long enough. (Online version in colour.)

Figure 4 shows four different data split strategies for a hypothetical time series of meteorological data. In order to enable an NN to forecast the next *k* time steps, one will generally feed an input vector of *l* past time steps to the network as input. In many DL applications, it is standard to draw random samples from a huge database of mutually independent data records (e.g. images) and arbitrarily assign these samples to the train, validation and test sets, respectively. This would correspond to figure 4*a*, where each drawn ‘slice’ has a length of one sample. However, as noted in §4, meteorological data constitutes a continuous time series with auto-correlation on different time scales. Therefore, randomly drawn samples would overlap and therefore no longer be independent. Consequently, results obtained with such a test set over-estimate the true generalization capability of the NN, because the test set contains information already used for training. When researching for this article, we found several studies on ML and DL for environmental data analysis, where this principle was violated and which therefore made overly optimistic conclusions concerning the capabilities of (often simple) NNs.

Another point of concern, which also has implications for the data preparation, is the multi-scale aspect of data in the time domain. While a typical forecast application considers time scales of a few hours to several days, there are longer-term quasi-periodic patterns, such as the El Niño Southern Oscillation (ENSO), and also continuous trends such as global warming. When training NNs with long-term data series (so that a sufficient number of samples becomes available), it is not trivial to find a good data split, which on the one hand fulfils the requirement of independence, while on the other hand allows the network to be trained on as many parts of the underlying data distribution as possible. For example, the model developer should ensure that all seasons are sampled appropriately, and, when using multi-year data, that the training data contains different phases of ENSO as one example out of many other oscillations.

To solve these issues, we propose a random block sampling strategy (figure 4*b*–*d*), where the train, validation and test sets all contain several coherent blocks of length *L*, and *L* is much larger than the auto-correlation time. Multiple runs of this random block sampling should be carried out to assess the robustness of the results.

The second aspect which we would like to discuss in this section concerns model evaluation. While it is relatively straightforward to evaluate the success of an image classification (e.g. [8,69]) or a video prediction task (cf. [12,79]), the evaluation metrics used in these studies (e.g. MSE or Peak Signal to Noise Ratio, PSNR) are usually not appropriate for weather and climate applications. Quantifying the success of a weather prediction model is highly non-trivial and an area of active research. Meteorological centres have developed a plethora of scores and skill scores over the last decades, which elucidate different aspects of weather forecast quality (cf. [21,91,114]). In addition to verification methods based on point by point comparisons (e.g. [115]), various methods have been introduced to account for the intrinsic spatial and temporal correlation in meteorological datasets (e.g. [116–118]). Other verification metrics also account for the stochastic nature of meteorological quantities by estimating probabilities of binary events, such as rain, no-rain [119]. Evaluating spatiotemporal patterns, for example, precipitation forecasts, with the help of radar data is particularly challenging due to the double penalty problem (cf. [116,120]). Indeed, verification of precipitation forecasts is still a hot topic in the meteorological community [121]. The evaluation of extreme events suffers from the ‘forecaster’s dilemma’, which discredits skilful forecasts when they are evaluated only under the condition that an extreme event occurred. This conditioning on outcomes and observations violates the theoretical assumptions of forecast verification methods [21,122].

## 6. Physical constraints and consistency

As has been demonstrated in other application areas of ML (e.g. [123,124]), NNs can be prone to learning spurious relationships in data. A purely data-driven model for weather forecasting might fail to adhere to the underlying physical principles and thus generate false forecasts as it lacks understanding of the fact that every atmospheric process obeys physical laws described in terms of conservation of momentum, enthalpy and mass. The incorporation of physical laws in NNs is becoming a vibrant area of research (e.g. [125–128]) and is now often denoted as scientific ML.

One of the first studies to demonstrate that physical constraints can efficiently reduce systematic biases in lake temperature predictions, while at the same time enhancing generalization capability, was Karpatne *et al.* [129]. They included numerical model results as constraints for sparse observations and added a loss term to punish non-physical behaviour of the DL model. De Bézenac *et al.* [127] embedded an advection diffusion equation in their loss function to predict sea surface temperatures and thereby improved the predictive capabilities of their model. Le Guen *et al.* [76] introduced a new two-stream model and a recurrent cell, which is based on concepts from DA. The cell includes a physical predictor and a Kalman filter in order to assimilate the inputs. Effectively, this leads to the encoding of physical laws in the latent space of their model. Other approaches to introduce physical constraints into the latent space of DL models are the adversarial autoencoder by Makhzani *et al.* [130] and the non-parametric Bayesian method by Goyal *et al.* [131].

It may be useful to reflect on the potential and necessity of physically constraining DL models from an abstract point of view. In spite of their complexity and dimensionality, DL models still adhere to the fundamental principles of (data-driven) statistical modelling. This implies that there must be some rules in place to constrain the future, because otherwise extrapolation will be unbound. Classical statistical modelling tries to strike a balance between a sufficiently explicit formulation of the time-dependent system evolution and the remaining degrees of freedom to accommodate the intrinsic variability of the data. For example, to fit hourly temperature observations, a classical statistical model will usually include (at least) two periodic terms to capture the diurnal and seasonal cycles. In addition, there may be terms to describe correlations of temperature with other variables. On the other hand, the statistician will avoid over-fitting the data by adding too many terms into the statistical model.

Even though in DL one expects the NN to learn many of the inherent data relations by itself, the system nevertheless must get some guidance to be able to identify meaningful patterns. Knowingly or not, the researcher always imposes constraints on the NN, for example through data selection and choice of NN architecture. NNs learn faster when patterns are clearly visible in the data. However, with meteorological data the most obvious patterns are usually the least interesting ones, therefore it makes sense to let the NN know in advance that such patterns will occur. A similar argument applies to physical constraints: if the NN is forced, for example to conserve mass, it will not need to waste parameters and training cycles on learning this rule and can instead concentrate on analysing relevant and less obvious relations. Many meteorological studies show that it is often necessary to pre-select or filter data and build a good statistical model before meaningful relations become apparent. It is, therefore, likely that an end-to-end DL weather forecast system will only be successful if it contains at least some *a priori* knowledge in the form of engineered data features and physical constraints. Just how much of this is needed remains to be seen.

Newcomers to the field of NWP often think that such numerical simulation models are inherently self-consistent, because they are based on a well-defined set of differential equations. However, the discretization of partial differential equations describing the flow dynamics in NWP models is not always fully mass or energy conserving, and parameterization schemes, which are needed to incorporate the effects of unresolved sub-grid scale processes on the grid-scale variables, may lead to spurious competition between grid-scale and sub-grid-scale processes, for example in cloud schemes [132,133]. Furthermore, there can be a grey zone between different parameterizations describing related aspects, such as the classical distinction of shallow and deep convection. Also, physically related parameterization schemes may be derived from different empirical data. Furthermore, internal consistency of classical NWP no longer applies if one considers the entire NWP workflow, i.e. if statistical models are used to post-process the model output, remove biases and apply other, non-physical corrections to the model forecasts. This discussion does not intend to devalue classical NWP, but it should inspire some reflection on the exact meaning and the value of consistency in the weather forecasting and DL communities. An end-to-end DL weather forecast system will generate consistency among forecast products only to the extent that this is already embedded in the data, unless the system will be governed by physical constraints as discussed above. To what extent consistency is needed to obtain a ‘good’ forecast will be a worthwhile question to study as it may deepen the understanding of the problem at hand and the potential which DL can bring to weather forecasting.

## 7. Uncertainty estimation

The final discussion point of this article concerns the estimation of forecast uncertainty. Owing to increased computer power, it has become possible in recent years to produce ensemble forecasts operationally. Ensemble approaches have also been introduced in DA (see §2). In a nutshell, ensemble forecasts aim to estimate the probability density function of the forecast variables. Ensembles are most often generated by varying the initial conditions of the model simulation, but there are also attempts to sample the parameter space of empirical model parameterizations.

In the field of DL research ensemble methods are used less often because they are computationally expensive. Statistical concepts such as Gaussian process (GP), and probabilistic graphical models (PGM) excel at probabilistic inference and uncertainty estimation. However, these methods do not scale well for high-dimensional and high-volume data [134–136]. Therefore, Bayesian deep learning (BDL) has been developed and applied across several scientific and engineering domains, for example in medical diagnosis [137] or autonomous driving [138]. In essence, these methods estimate a probability density function of the DL model parameters. As side effects BDL increases the robustness against over-fitting and allows training of the NN from relatively small sample sizes [139]. Modern BDL methods include variational inference, Markov Chain Monte Carlo (MCMC) sampling [136], and Monte Carlo dropout [140].

Some recent studies explored the BDL concept for weather forecasting applications. A model built on GRU and 3D CNN, along with variational Bayesian inference for estimating posterior parameter distributions, has been presented by Liu *et al.* [141] for probabilistic wind speed forecasting of up to 3 h. A study from Vandal *et al*. [92] demonstrates the use of BDL to capture the uncertainty from observation data and unknown model parameters in the context of statistical downscaling of precipitation forecasts. These are relevant contributions, but a lot remains to be done before the uncertainty of DL weather forecasts can be assessed at a level similar to current NWP ensemble systems.

## 8. Conclusion

In this article, we discussed the potential of modern DL approaches to develop purely data-driven end-to-end weather forecast applications. While there have been some stunning success stories from DL applications in other fields and initial attempts were made to apply DL to meteorological data, this research is still in its infancy. As we argue in §4, there are specific properties of weather data which require the development of new approaches beyond the classical concepts from computer vision, speech recognition, and other typical ML tasks. Even though DL solutions for many of these issues are being developed, there is no DL method up to now which can deal with all of these issues concurrently as it would be required in a complete weather forecast system.

We expect that the field of ML in weather and climate science will grow rapidly in the coming years as more and more sophisticated ML architectures are becoming available and can easily be deployed on modern computer systems. What is largely missing in the field of meteorological DL are benchmark datasets with a specification of appropriate baseline scores and software frameworks which make it easy for the DL community to adopt a meteorological problem and try out different approaches. One notable exception is Weatherbench [142]. Such benchmark datasets and frameworks are well established in the ML community (e.g. MNIST [143] or ImageNet [144]) and they contributed substantially to the rapid pace of DL developments in application areas such as image recognition, video prediction, speech recognition, gaming and robotics. While a lot of meteorological data is freely available from weather centres and research institutions, proper use of these data requires some knowledge about Earth system science and the data formats and tools, which are used by the environmental research community. It might help if tools for reading and working with these datasets were integrated in major ML frameworks.

When reflecting on the ultimate goal of replacing computationally expensive NWP models with DL algorithms, it is important to reconsider the objectives of weather forecasting and carefully define the requirements, which must be met by any potential alternative method. Certain criteria, which we now consider essential for a ‘good’ weather forecast, may in fact be conceptions, which are resulting from our experiences with numerical models, and they may not be applicable to forecasting systems based on DL. One particular aspect in this regard is self-consistency of forecast results, which is often taken for granted by numerical modellers, even though it is not strictly fulfilled in current NWP forecast systems. In this article, we consciously propose thinking about a replacement of the entire core NWP workflow including the DA, numerical modelling, and output processing, because the task of weather forecasting can then be described as a huge Big Data problem of mapping a plethora of Earth system observations onto a well-defined set of specific end-user weather forecast products. Seen in this way, the problem of weather forecasting is more amenable to DL methods than a replacement of the actual NWP model itself with its grid structure, operator concepts etc. which are tied to the very concept of classical numerical modelling.

We expect that the success of DL weather forecast applications will hinge on the consideration of physical constraints in the NN design. Taken to the extreme, portions or variants of current numerical models could eventually end up as regulators in the latent space of deep neural weather forecasting networks. So, to answer the question posed in the title of this article, we can only say that there might be potential for end-to-end DL weather forecast applications to produce equal or better quality forecasts for specific end-user demands, especially if these systems can exploit small-scale patterns in the observational data which are not resolved in the traditional NWP model chain. Whether DL will evolve enough to replace most or all of the current NWP systems cannot be answered at this point.

## Data accessibility

This article has no additional data.

## References

[1] Lynch P 2008 The origins of computer weather prediction and climate modeling. J. Comput. Phys. 227, 3431–3444. (10.1016/j.jcp.2007.02.034)

[2] Bauer P, Thorpe A, Brunet G 2015 The quiet revolution of numerical weather prediction. Nature 525, 47–55. (10.1038/nature14956) 26333465

[3] Schmidhuber J 2015 Deep learning in neural networks: an overview. Neural Netw. 61, 85–117. (10.1016/j.neunet.2014.09.003) 25462637

[4] McCulloch WS, Pitts W 1943 A logical calculus of the ideas immanent in nervous activity. Bull. Math. Biophys. 5, 115–133. (10.1007/BF02478259) 2185863

[5] Linnainmaa S 1970 The representation of the cumulative rounding error of an algorithm as a taylor expansion of the local rounding errors. *Master’s Thesis (in Finnish), Univ. Helsinki*.

[6] LeCun Y 1988 A theoretical framework for back-propagation. In *Proc. of the 1988 Connectionist Models Summer School, CMU, Pittsburg, PA* (eds D Touretzky, G Hinton, T Sejnowski), pp. 21–28. Morgan Kaufmann.

[7] Rumelhart DE, Hinton GE, Williams RJ 1986 *Learning Internal Representations by Error Propagation*, pp. 318–362. Cambridge, MA: MIT Press.

[8] Krizhevsky A, Sutskever I, Hinton GE 2012 Imagenet classification with deep convolutional neural networks. In *Advances in Neural Information Processing Systems 25* (eds F Pereira, CJC Burges, L Bottou, KQ Weinberger), pp. 1097–1105. Curran Associates, Inc.

[9] Amodei D *et al.* 2016 Deep speech 2: end-to-end speech recognition in english and mandarin. In *Proc. of The 33rd Int. Conf. on Machine Learning*, volume 48 of *Proc. of Machine Learning Research*, pp. 173–182. New York, NY: PMLR. (https://arxiv.org/abs/1512.02595).

[10] Silver D *et al.* 2016 Mastering the game of go with deep neural networks and tree search. Nature 529, 484–489. (10.1038/nature16961) 26819042

[11] Oh J, Guo X, Lee H, Lewis RL, Singh S 2015 Action-conditional video prediction using deep networks in atari games. In *Advances in Neural Information Processing Systems 28* (eds C Cortes, ND Lawrence, DD Lee, M Sugiyama, R Garnett), pp. 2863–2871. Curran Associates, Inc.

[12] Mathieu M, Couprie C, LeCun Y 2015 Deep multi-scale video prediction beyond mean square error. (http://arxiv.org/abs/1511.05440).

[13] Lee AX, Zhang R, Ebert F, Abbeel P, Finn C, Levine S 2018 Stochastic adversarial video prediction. (http://arxiv.org/abs/1804.01523).

[14] Reichstein M, Camps-Valls G, Stevens B, Jung M, Denzler J, Carvalhais N 2019 Deep learning and process understanding for data-driven earth system science. Nature 566, 195–204. (10.1038/s41586-019-0912-1) 30760912

[15] Jiang Z, Jia Q, Guan X 2017 Review of wind power forecasting methods: From multi-spatial and temporal perspective. In *2017 36th Chinese Control Conference (CCC)*, pp. 10 576–10 583 (10.23919/chicc.2017.8029042)

[16] Grover A, Kapoor A, Horvitz E 2015 A deep hybrid model for weather forecasting. In *Proc. of the 21th ACM SIGKDD Int. Conf. on Knowledge Discovery and Data Mining*, KDD ’15, pp. 379–386. New York, NY: Association for Computing Machinery. (10.1145/2783258.2783275)

[17] Thépaut JN, Andersson E 2010 *The global observing system*, pp. 263–281. Berlin, Heidelberg: Springer. (10.1007/978-3-540-74703-1_10)

[18] Bannister RN 2017 A review of operational methods of variational and ensemble-variational data assimilation: ensemble-variational data assimilation. Q. J. R. Meteorol. Soc. 143, 607–633. (10.1002/qj.2982)

[19] Al-Yahyai S, Charabi Y, Gastli A 2010 Review of the use of numerical weather prediction (NWP) models for wind energy assessment. Renew. Sustain. Energy Rev. 14, 3192–3198. (10.1016/j.rser.2010.07.001)

[20] Pelland S, Galanis G, Kallos G 2013 Solar and photovoltaic forecasting through post-processing of the Global Environmental Multiscale numerical weather prediction model: solar and photovoltaic forecasting. Prog. Photovoltaics Res. Appl. 21, 284–296. (10.1002/pip.1180)

[21] Haiden T, Janousek M, Vitart F, Ferranti L, Prates F 2019 Evaluation of ECMWF forecasts, including the 2019 upgrade. (10.21957/mlvapkke)

[22] Chen JH *et al.* 2019 Advancements in hurricane prediction with noaa’s next-generation forecast system. Geophys. Res. Lett. 46, 4495–4501. (10.1029/2019GL082410)

[23] Geer AJ *et al.* 2017 The growing impact of satellite observations sensitive to humidity, cloud and precipitation. Q. J. R. Meteorol. Soc. 143, 3189–3206. (10.1002/qj.3172)

[24] Lange H, Janjic Pfander T 2015 Assimilation of mode-S EHS aircraft observations in cosmo-kenda. Mon. Weather Rev. 144, 151221072928005 (10.1175/MWR-D-15-0112.1)

[25] Schraff C, Reich H, Rhodin A, Schomburg A, Stephan K, Periàñez A, Potthast R 2016 Kilometre-scale ensemble data assimilation for the cosmo model (kenda). Q. J. R. Meteorol. Soc. 142, 1453–1472. (10.1002/qj.2748)

[26] Wang H, Sun J, Zhang X, Huang XY, Auligné T 2013 Radar data assimilation with WRF 4D-Var. Part I: system development and preliminary testing. Mon. Weather Rev. 141, 2224–2244. (10.1175/MWR-D-12-00168.1)

[27] Vukićević T, Bao JW 1998 The effect of linearization errors on 4dvar data assimilation. Mon. Weather Rev. 126, 1695–1706. (10.1175/1520-0493(1998)126¡1695:TEOLEO¿2.0.CO;2)

[28] Steele-Dunne S, Entekhabi D 2005 An ensemble-based reanalysis approach to land data assimilation. Water Resour. Res. 41 (10.1029/2004WR003449)

[29] Zhang F, Zhang M, Hansen JA 2009 Coupling ensemble kalman filter with four-dimensional variational data assimilation. Adv. Atmos. Sci. 26, 1–8. (10.1007/s00376-009-0001-8)

[30] Zängl G, Reinert D, Rípodas P, Baldauf M 2015 The icon (icosahedral non-hydrostatic) modelling framework of DWD and MPI-M: description of the non-hydrostatic dynamical core. Q. J. R. Meteorol. Soc. 141, 563–579. (10.1002/qj.2378)

[31] Tomita H, Satoh M 2004 A new dynamical framework of nonhydrostatic global model using the icosahedral grid. Fluid Dyn. Res. 34, 357–400. (10.1016/j.fluiddyn.2004.03.003)

[32] Ullrich PA, Jablonowski C 2012 Mcore: A non-hydrostatic atmospheric dynamical core utilizing high-order finite-volume methods. J. Comput. Phys. 231, 5078–5108. (10.1016/j.jcp.2012.04.024)

[33] Ringler TD, Thuburn J, Klemp JB, Skamarock WC 2010 A unified approach to energy conservation and potential vorticity dynamics for arbitrarily-structured c-grids. J. Comput. Phys. 229, 3065–3090. (10.1016/j.jcp.2009.12.007)

[34] Wood N *et al.* 2014 An inherently mass-conserving semi-implicit semi-lagrangian discretization of the deep-atmosphere global non-hydrostatic equations. Q. J. R. Meteorol. Soc. 140, 1505–1520. (10.1002/qj.2235)

[35] Gassmann A 2018 Discretization of generalized coriolis and friction terms on the deformed hexagonal c-grid. Q. J. R. Meteorol. Soc. 144, 2038–2053. (10.1002/qj.3294)

[36] Bechtold P, Semane N, Lopez P, Chaboureau JP, Beljaars A, Bormann N 2014 Representing equilibrium and nonequilibrium convection in large-scale models. J. Atmos. Sci. 71, 734–753. (10.1175/JAS-D-13-0163.1)

[37] Kwon YC, Hong SY 2017 A mass-flux cumulus parameterization scheme across gray-zone resolutions. Mon. Weather Rev. 145, 583–598. (10.1175/MWR-D-16-0034.1)

[38] Neggers RAJ, Köhler M, Beljaars ACM 2009 A dual mass flux framework for boundary layer convection. Part I: transport. J. Atmos. Sci. 66, 1465–1487. (10.1175/2008JAS2635.1)

[39] Seifert A, Beheng KD 2006 A two-moment cloud microphysics parameterization for mixed-phase clouds. Part 1: model description. Meteorol. Atmos. Phys. 92, 45–66. (10.1007/s00703-005-0112-4)

[40] LeCun Y, Haffner P, Bottou L, Bengio Y 1999 *Object Recognition with Gradient-Based Learning*, pp. 319–345. Berlin, Heidelberg: Springer. (10.1007/3-540-46805-6_19)

[41] Zhu XX, Tuia D, Mou L, Xia GS, Zhang L, Xu F, Fraundorfer F 2017 Deep learning in remote sensing: a comprehensive review and list of resources. IEEE Geosci. Remote Sensing Mag. 5, 8–36. (10.1109/MGRS.2017.2762307)

[42] Gagne II DJ, Haupt SE, Nychka DW, Thompson G 2019 Interpretable deep learning for spatial analysis of severe hailstorms. Mon. Weather Rev. 147, 2827–2845. (10.1175/MWR-D-18-0316.1)

[43] Hochreiter S, Schmidhuber J 1997 Long short-term memory. Neural Comput. 9, 1735–1780. (10.1162/neco.1997.9.8.1735) 9377276

[44] Gers FA, Schmidhuber J 2000 Recurrent nets that time and count. In *Proc. of the IEEE-INNS-ENNS Int. Joint Conference on Neural Networks. IJCNN 2000. Neural Computing: New Challenges and Perspectives for the New Millennium*, volume 3, pp. 189–194. Como, Italy, Italy. (10.1109/IJCNN.2000.861302)

[45] Chung J, Gülçehre Ç, Cho K, Bengio Y 2014 Empirical evaluation of gated recurrent neural networks on sequence modeling. *CoRR***abs/1412.3555**. (http://arxiv.org/abs/1412.3555).

[46] Xingjian S, Chen Z, Wang H, Yeung DY, Wong WK, Woo Wc 2015 Convolutional LSTM network: A machine learning approach for precipitation nowcasting. In *Advances in neural information processing systems*, pp. 802–810.

[47] Kingma DP, Welling M 2014 Auto-Encoding Variational Bayes. *CoRR*. (http://arxiv.org/abs/1312.6114).

[48] Goodfellow I, Pouget-Abadie J, Mirza M, Xu B, Warde-Farley D, Ozair S, Courville A, Bengio Y 2014 Generative adversarial nets. In *Advances in Neural Information Processing Systems*, pp. 2672–2680. Curran Associates, Inc.

[49] Isola P, Zhu JY, Zhou T, Efros AA 2017 Image-to-image translation with conditional adversarial networks. In *Proc. of the IEEE Conf. on Ccomputer Vision and Pattern Recognition*, Vol. 1, pp. 5967–5976. (10.1109/CVPR.2017.632)

[50] Johnson J, Alahi A, Fei-Fei L 2016 Perceptual losses for real-time style transfer and super-resolution. *Lecture Notes in Computer Science*, pp. 694–711. (10.1007/978-3-319-46475-6_43)

[51] Pathak D, Krahenbuhl P, Donahue J, Darrell T, Efros AA 2016 Context encoders: Feature learning by inpainting. In *2016 IEEE Conf. on Computer Vision and Pattern Recognition (CVPR)*, pp. 2536–2544. Los Alamitos, CA, USA: IEEE Computer Society. (10.1109/CVPR.2016.278)

[52] Zhang H, Sindagi V, Patel VM 2019 Image de-raining using a conditional generative adversarial network. *IEEE Transactions on Circuits and Systems for Video Technology* p. 1–1. (10.1109/tcsvt.2019.2920407)

[53] Reed S, Akata Z, Yan X, Logeswaran L, Schiele B, Lee H 2016 Generative adversarial text to image synthesis. In *Proc. of The 33rd Int. Conf. on Machine Learning* (eds MF Balcan, KQ Weinberger), volume 48 of *Proc. of Machine Learning Research*, pp. 1060–1069. New York, NY: PMLR.

[54] Li C, Wand M 2016 Precomputed real-time texture synthesis with markovian generative adversarial networks. *Lecture Notes in Computer Science*, pp. 702–716. (10.1007/978-3-319-46487-9_43)

[55] Kingma DP, Welling M 2019 An introduction to variational autoencoders. (http://arxiv.org/abs/1906.02691).

[56] Gulrajani I, Kumar K, Ahmed F, Taiga AA, Visin F, Vazquez D, Courville A 2016 Pixelvae: A latent variable model for natural images. (http://arxiv.org/abs/1611.05013).

[57] Radford A, Metz L, Chintala S 2015 Unsupervised representation learning with deep convolutional generative adversarial networks. (http://arxiv.org/abs/1511.06434).

[58] Shaham TR, Dekel T, Michaeli T 2019 SinGAN: Learning a generative model from a single natural image. *2019 IEEE/CVF Int. Conf. on Computer Vision (ICCV)*. (10.1109/iccv.2019.00467)

[59] Schizas CN, Michaelides S, Pattichis CS, Livesay RR 1991 Artificial neural networks in forecasting minimum temperature (weather). In *Second Int. Conf. on Artificial Neural Networks*, volume 349, pp. 112–114. Bournemouth Int. CTR, Bournemouth, England: IEEE.

[60] Comrie AC 1997 Comparing neural networks and regression models for ozone forecasting. J. Air Waste Manage. Assoc. 47, 653–663. (10.1080/10473289.1997.10463925)

[61] Hall T, Brooks HE, Doswell III CA 1999 Precipitation forecasting using a neural network. Weather Forecast. 14, 338–345. (10.1175/1520-0434(1999)014¡0338:PFUANN¿2.0.CO;2)

[62] Krasnopolsky VM, Lin Y 2012 A neural network nonlinear multimodel ensemble to improve precipitation forecasts over continental US. Adv. Meteorol. 2012, 1–11. (10.1155/2012/649450)

[63] Rasp S, Lerch S 2018 Neural networks for postprocessing ensemble weather forecasts. Mon. Weather Rev. 146, 3885–3900. (10.1175/mwr-d-18-0187.1)

[64] Krasnopolsky VM, Chalikov DV, Tolman HL 2002 A neural network technique to improve computational efficiency of numerical oceanic models. Ocean Modelling 4, 363–383. (10.1016/S1463-5003(02)00010-0)

[65] Krasnopolsky VM, Fox-Rabinovitz MS 2006 Complex hybrid models combining deterministic and machine learning components for numerical climate modeling and weather prediction. Neural Netw. 19, 122–134. (10.1016/j.neunet.2006.01.002) 16527454

[66] Shi Y, Li Y, Liu J, Liu X, Murphey YL 2018 Weather recognition based on edge deterioration and convolutional neural networks. In *2018 24th Int. Conf. on Pattern Recognition (ICPR)*, pp. 2438–2443. IEEE. (10.1109/ICPR.2018.8546085)

[67] Simonyan K, Zisserman A 2014 Very deep convolutional networks for large-scale image recognition. (http://arxiv.org/abs/1409.1556).

[68] Pothineni D, Oswald MR, Poland J, Pollefeys M 2019 Kloudnet: Deep learning for sky image analysis and irradiance forecasting. In *Pattern Recognition* (eds T Brox, A Bruhn, M Fritz), pp. 535–551. Cham: Springer International Publishing. (10.1007/978-3-030--12939-2_37)

[69] He K, Zhang X, Ren S, Sun J 2016 Deep residual learning for image recognition. In *2016 IEEE Conf. on Computer Vision and Pattern Recognition (CVPR)*, pp. 770–778. Las Vegas, NV: IEEE. (10.1109/CVPR.2016.90)

[70] Wen H, Du Y, Chen X, Lim E, Wen H, Jiang L, Xiang W 2020 Deep learning-based multi-step solar forecasting for PV ramp-rate control using sky images. IEEE Trans. Ind. Inf. 17, 1397–1406. (10.1109/TII.2020.2987916)

[71] Szegedy C, Liu W, Jia Y, Sermanet P, Reed S, Anguelov D, Erhan D, Vanhoucke V, Rabinovich A 2015 Going deeper with convolutions. In *The IEEE Conf. on Computer Vision and Pattern Recognition (CVPR2015)*. (10.1109/CVPR.2015.7298594)

[72] Kleinert F, Leufen LH, Schultz MG 2021 IntelliO3-ts v1.0: a neural network approach to predict near-surface ozone concentrations in Germany. Geosci. Model Dev. 14, 1–25. (10.5194/gmd-14-1-2021)

[73] Bengio Y, Simard P, Frasconi P 1994 Learning long-term dependencies with gradient descent is difficult. IEEE Trans. Neural Netw. 5, 157–166. (10.1109/72.279181) 18267787

[74] Gómez P, Nebot A, Ribeiro S, Alquézar R, Mugica F, Wotawa F 2003 Local maximum ozone concentration prediction using soft computing methodologies. Syst. Anal. Model. Simul. 43, 1011–1031. (10.1080/0232929031000081244)

[75] Qing X, Niu Y 2018 Hourly day-ahead solar irradiance prediction using weather forecasts by LSTM. Energy 148, 461–468. (10.1016/j.energy.2018.01.177)

[76] Le Guen V, Thome N 2020 Disentangling physical dynamics from unknown factors for unsupervised video prediction. (http://arxiv.org/abs/2003.01460). 10.1109/TPAMI.2022.3152862 35201980

[77] Smolensky P 1986 *Chapter 6: Information Processing in Dynamical Systems: Foundations of Harmony Theory*, pp. 194–281. Cambridge, MA: MIT Press.

[78] Hinton GE, Salakhutdinov RR 2006 Reducing the dimensionality of data with neural networks. Science 313, 504–507. (10.1126/science.1127647) 16873662

[79] Xiong W, Luo W, Ma L, Liu W, Luo J 2018 Learning to generate time-lapse videos using multi-stage dynamic generative adversarial networks. In *2018 IEEE/CVF Conf. on Computer Vision and Pattern Recognition*. IEEE. (10.1109/cvpr.2018.00251)

[80] Schmidt VH, Alghali M, Sankaran K, Yuan T, Bengio Y 2020 Modeling cloud reflectance fields using conditional generative adversarial networks. *arXiv: Atmospheric and Oceanic Physics*.

[81] Wang Y, Long M, Wang J, Gao Z, Yu PS 2017 PredRNN: recurrent neural networks for predictive learning using spatiotemporal LSTMs. In *Proc. of the 31st Int. Conf. on Neural Information Processing Systems (NIPS 2017)*, pp. 879–888.

[82] Wang Y, Zhang J, Zhu H, Long M, Wang J, Yu PS 2019 Memory in Memory: a predictive neural network for learning higher-order non-stationarity from spatiotemporal dynamics. In *The IEEE Conf. on Computer Vision and Pattern Recognition (CVPR)*. (10.1109/CVPR.2019.00937)

[83] Zhou Y, Wang H, Xu F, Jin YQ 2016 Polarimetric SAR image classification using deep convolutional neural networks. IEEE Geosci. Remote Sens. Lett. 13, 1935–1939. (10.1109/LGRS.2016.2618840)

[84] Denby L 2020 Discovering the importance of mesoscale cloud organization through unsupervised classification. GeoRL 47, e85190 (10.1029/2019GL085190)

[85] Xu Z, Du J, Wang J, Jiang C, Ren Y 2019 Satellite image prediction relying on gan and lstm neural networks. In *ICC 2019-2019 IEEE Int. Conf. on Communications (ICC)*, pp. 1–6. IEEE. (10.1109/ICC.2019.8761462)

[86] Dueben PD, Bauer P 2018 Challenges and design choices for global weather and climate models based on machine learning. Geoscientific Model Dev. 11, 3999–4009. (10.5194/gmd-11-3999-2018)

[87] Wandel N, Weinmann M, Klein R 2020 Unsupervised deep learning of incompressible fluid dynamics. http://arxiv.org/abs/2006.08762.

[88] Parisi GI, Kemker R, Part JL, Kanan C, Wermter S 2018 Continual lifelong learning with neural networks: a review. http://arxiv.org/abs/1802.07569v4. 10.1016/j.neunet.2019.01.012 30780045

[89] Silver DL, Bennett KP 2008 Guest editor’s introduction: special issue on inductive transfer learning. Mach. Learn. 73, 215–220. (10.1007/s10994-008-5087-1)

[90] Schultz M, Pleiter D, Bauer P eds. 2019, 2018. Extreme Data Workshop 2018, Forschungszentrum Jülich, 18–19 September 2018, ISBN 978-3-95806-392-1, Schriften des Forschungszentrums Jülich, IAS Series 40.

[91] Wilks DS 2006 Statistical methods in the atmospheric sciences, vol. 91, 2nd edn International geophysics series Amsterdam; The Netherlands: Academic Press.

[92] Vandal T, Kodra E, Dy J, Ganguly S, Nemani R, Ganguly AR 2018 Quantifying uncertainty in discrete-continuous and skewed data with Bayesian deep learning. In *Proc. of the 24th ACM SIGKDD Int. Conf. on Knowledge Discovery & Data Mining*, KDD-18, p. 2377–2386. New York, NY: Association for Computing Machinery. (10.1145/3219819.3219996)

[93] Orlanski I 1975 A rational subdivision of scales for atmospheric processes. Bull. Am. Meteorol. Soc. 56, 527–530.

[94] Semmler T, Jung T, Serrar S 2016 Fast atmospheric response to a sudden thinning of Arctic sea ice. Clim. Dyn. 46, 1015–1025. (10.1007/s00382-015-2629-7)

[95] Partal T, Kişi Ö 2007 Wavelet and neuro-fuzzy conjunction model for precipitation forecasting. J. Hydrol. 342, 199–212. (10.1016/j.jhydrol.2007.05.026)

[96] Ramana RV, Krishna B, Kumar S, Pandey N 2013 Monthly rainfall prediction using wavelet neural network analysis. Water Resour. Manage 27, 3697–3711. (10.1007/s11269-013-0374-4)

[97] Solgi A, Nourani V, Pourhaghi A 2014 Forecasting daily precipitation using hybrid model of wavelet-artificial neural network and comparison with adaptive neurofuzzy inference system (case study: Verayneh station, Nahavand). Adv. Civil Eng. 2014, 1–12. (10.1155/2014/279368)

[98] Kisi O, Cimen M 2012 Precipitation forecasting by using wavelet-support vector machine conjunction model. Eng. Appl. Artif. Intell. 25, 783–792. (10.1016/j.engappai.2011.11.003)

[99] Ziyin L, Hartwig T, Ueda M 2020 Neural networks fail to learn periodic functions and how to fix it. http://arxiv.org/abs/2006.08195.

[100] Cirstea RG, Micu DV, Muresan GM, Guo C, Yang B 2018 Correlated time series forecasting using deep neural networks: a summary of results. http://arxiv.org/abs/1808.09794.

[101] Unwetterklimatologie: Starkregen. www.dwd.de/DE/leistungen/unwetterklima/starkregen/starkregen.html. (accessed 30 April 2020).

[102] Vandal T, Kodra E, Ganguly AR 2019 Intercomparison of machine learning methods for statistical downscaling: the case of daily and extreme precipitation. Theor. Appl. Climatol. 137, 557–570. (10.1007/s00704-018-2613-3)

[103] O’Gorman PA, Dwyer JG 2018 Using machine learning to parameterize moist convection: potential for modeling of climate, climate change, and extreme events. J. Adv. Model. Earth Syst. 10, 2548–2563. (10.1029/2018MS001351)

[104] He H, Ma Y eds. 2013 Imbalanced learning: foundations, algorithms, and applications, 1st edn Hoboken, NJ: Wiley (10.1002/9781118646106)

[105] Torgo L, Ribeiro RP, Pfahringer B, Branco P 2013 Smote for regression. In *Progress in Artificial Intelligence* (eds L Correia, LP Reis, J Cascalho), pp. 378–389. Berlin, Heidelberg: Springer. (10.1007/978-3-642-40669-0_33)

[106] Lake BM, Baroni M 2017 Generalization without systematicity: on the compositional skills of sequence-to-sequence recurrent networks.

[107] Li Fei-Fei, Fergus R, Perona P 2006 One-shot learning of object categories. IEEE Trans. Pattern. Anal. 28, 594–611. (10.1109/TPAMI.2006.79) 16566508

[108] Lake BM, Salakhutdinov R, Tenenbaum JB 2015 Human-level concept learning through probabilistic program induction. Science 350, 1332–1338. (10.1126/science.aab3050) 26659050

[109] Smieja M, Łukasz Struski, Tabor J, Zieliński B, Spurek P 2018 Processing of missing data by neural networks. http://arxiv.org/abs/1805.07405.

[110] žliobaitė I 2010 Learning under concept drift: an overview. http://arxiv.org/abs/1010.4784.

[111] Lu J, Liu A, Dong F, Gu F, Gama J, Zhang G 2018 Learning under concept drift: a review. IEEE Trans. Knowl. Data Eng. 31, 2346–2363. (10.1109/tkde.2018.2876857)

[112] Barth A, Alvera-Azcárate A, Licer M, Beckers JM 2020 Dincae 1.0: a convolutional neural network with error estimates to reconstruct sea surface temperature satellite observations. Geoscientific Model Dev. 13, 1609–1622. (10.5194/gmd-13-1609-2020)

[113] Goodfellow I, Bengio Y, Courville A 2016 Deep learning. Cambridge, MA: MIT Press.

[114] Casati B *et al.* 2008 Forecast verification: current status and future directions. Meteorol. Appl. 15, 3–18. (10.1002/met.52)

[115] Murphy AH, Epstein ES 1989 Skill scores and correlation coefficients in model verification. Mon. Weather Rev. 117, 572–582. (10.1175/1520-0493(1989)117¡0572:SSACCI¿2.0.CO;2)

[116] Ebert EE 2008 Fuzzy verification of high-resolution gridded forecasts: a review and proposed framework. Meteorol. Appl. 15, 51–64. (10.1002/met.25)

[117] Weniger M, Kapp F, Friederichs P 2017 Spatial verification using wavelet transforms: a review: spatial verification using wavelet transforms. Q. J. R. Meteorol. Soc. 143, 120–136. (10.1002/qj.2881)

[118] Buschow S, Pidstrigach J, Friederichs P 2019 Assessment of wavelet-based spatial verification by means of a stochastic precipitation model (wv_verif v0.1.0). Geoscientific Model Dev. 12, 3401–3418. (10.5194/gmd-12-3401-2019)

[119] Roberts NM, Lean HW 2008 Scale-selective verification of rainfall accumulations from high-resolution forecasts of convective events. Mon. Weather Rev. 136, 78–97. (10.1175/2007MWR2123.1)

[120] Gilleland E, Ahijevych D, Brown BG, Casati B, Ebert EE 2009 Intercomparison of spatial forecast verification methods. Weather Forecast. 24, 1416–1430. (10.1175/2009WAF2222269.1)

[121] Dorninger M, Gilleland E, Casati B, Mittermaier MP, Ebert EE, Brown BG, Wilson LJ 2018 The setup of the MesoVICT project. Bull. Am. Meteorol. Soc. 99, 1887–1906. (10.1175/BAMS-D-17-0164.1)

[122] Lerch S, Thorarinsdottir TL, Ravazzolo F, Gneiting T 2017 Forecaster’s dilemma: extreme events and forecast evaluation. Stat. Sci. 32, 106–127. (10.1214/16-STS588)

[123] Lazer D, Kennedy R, King G, Vespignani A 2014 The parable of google flu: traps in big data analysis. Science 343, 1203–1205. (10.1126/science.1248506) 24626916

[124] Lapuschkin S, Wäldchen S, Binder A, Montavon G, Samek W, Müller KR 2019 Unmasking clever Hans predictors and assessing what machines really learn. Nat. Commun. 10, 1–8. (10.1038/s41467-019-08987-4) 30858366 PMC6411769

[125] Karpatne A, Atluri G, Faghmous JH, Steinbach M, Banerjee A, Ganguly A, Shekhar S, Samatova N, Kumar V 2017 Theory-guided data science: a new paradigm for scientific discovery from data. IEEE Trans. Knowl. Data Eng. 29, 2318–2331. (10.1109/TKDE.2017.2720168)

[126] Jia X, Willard J, Karpatne A, Read J, Zwart J, Steinbach M, Kumar V 2019 Physics guided rnns for modeling dynamical systems: a case study in simulating lake temperature profiles. In *Proc. of the 2019 SIAM Int. Conf. on Data Mining*, pp. 558–566. SIAM. (10.1137/1.9781611975673.63)

[127] de Bézenac E, Pajot A, Gallinari P 2019 Deep learning for physical processes: incorporating prior scientific knowledge. J. Stat. Mech: Theory Exp. 2019, 124009 (10.1088/1742-5468/ab3195)

[128] Raissi M, Perdikaris P, Karniadakis GE 2019 Physics-informed neural networks: a deep learning framework for solving forward and inverse problems involving nonlinear partial differential equations. J. Comput. Phys. 378, 686–707. (10.1016/j.jcp.2018.10.045)

[129] Karpatne A, Watkins W, Read J, Kumar V 2017 Physics-guided neural networks (PGNN): an application in lake temperature modeling. http://arxiv.org/abs/1710.11431

[130] Makhzani A, Shlens J, Jaitly N, Goodfellow I, Frey B 2015 Adversarial autoencoders. http://arxiv.org/abs/1511.05644

[131] Goyal P, Hu Z, Liang X, Wang C, Xing EP 2017 Nonparametric variational auto-encoders for hierarchical representation learning. In *Proc. of the IEEE Int. Conf. on Computer Vision*, pp. 5094–5102. (10.1109/ICCV.2017.545)

[132] Arakawa A, Wu CM 2013 A unified representation of deep moist convection in numerical modeling of the atmosphere. Part I. J. Atmos. Sci. 70, 1977–1992. (10.1175/JAS-D-12-0330.1)

[133] Wu CM, Arakawa A 2014 A unified representation of deep moist convection in numerical modeling of the atmosphere. Part II. J. Atmos. Sci. 71, 2089–2103. (10.1175/JAS-D-13-0382.1)

[134] Zhu Y, Zabaras N, Koutsourelakis PS, Perdikaris P 2019 Physics-constrained deep learning for high-dimensional surrogate modeling and uncertainty quantification without labeled data. J. Comput. Phys. 394, 56–81. (10.1016/j.jcp.2019.05.024)

[135] Wang H, Yeung DY 2016 Towards Bayesian deep learning: A survey. http://arxiv.org/abs/1604.01662

[136] Wang H, Yeung D 2016 Towards Bayesian deep learning: a framework and some existing methods. IEEE Trans. Knowl. Data Eng. 28, 3395–3408. (10.1109/TKDE.2016.2606428)

[137] Gal Y, Islam R, Ghahramani Z 2017 Deep Bayesian active learning with image data. In *Proc. of the 34th Int. Conf. on Machine Learning* (ed. D Precup, YW Teh), volume 70 of *Proc. of Machine Learning Research*, pp. 1183–1192. International Convention Centre, Sydney, Australia: PMLR. http://proceedings.mlr.press/v70/gal17a

[138] McAllister R, Gal Y, Kendall A, Van Der, Shah A, Cipolla R, Weller A 2017 Concrete problems for autonomous vehicle safety: Advantages of Bayesian deep learning. In *Proc. of the Twenty-Sixth Int. Joint Conf. on Artificial Intelligence*. International Joint Conferences on Artificial Intelligence, Inc. (10.24963/ijcai.2017/661)

[139] Gal Y 2016 Uncertainty in deep learning. *University of Cambridge* **1**. https://pdfs.semanticscholar.org/55cd/9e1bb7ce02cd2bb01b364e7b331fcc1ef2c7.pdf?_ga=2.240865606.499308263.1596635628-148943261.1587029296.

[140] Gal Y, Ghahramani Z 2016 Dropout as a bayesian approximation: Representing model uncertainty in deep learning. In *Proc. of The 33rd Int. Conf. on Machine Learning* (ed. MF Balcan, KQ Weinberger), volume 48 of *Proc. of Machine Learning Research*, pp. 1050–1059. New York, NY: PMLR. http://proceedings.mlr.press/v48/gal16.html

[141] Liu Y, Qin H, Zhang Z, Pei S, Jiang Z, Feng Z, Zhou J 2020 Probabilistic spatiotemporal wind speed forecasting based on a variational Bayesian deep learning model. Appl. Energy 260, 114259 (10.1016/j.apenergy.2019.114259)

[142] Rasp S, Dueben PD, Scher S, Weyn JA, Mouatadid S, Thuerey N 2020 Weatherbench: a benchmark dataset for data-driven weather forecasting. http://arxiv.org/abs/2002.00469.

[143] LeCun Y, Bottou L, Bengio Y, Haffner P 1998 Gradient-based learning applied to document recognition. Proc. IEEE 86, 2278–2324. (10.1109/5.726791)

[144] Russakovsky O *et al.* 2015 Imagenet large scale visual recognition challenge. Int. J. Comput. Vision 115, 211–252. (10.1007/s11263-015-0816-y)

